# Personal

**Published:** 1888-09

**Authors:** 


### Personal.

DR. JOSEPH C. GREENE AND DR. S. S. GREENE, of this city, will start on Monday, September 3d, for a tour around the world. The two physicians have laid out an extended route, and will first visit San Francisco, then Yokohama, Hong Kong, Ceylon, Calcutta, Singapore, Delhi, and while in India will hunt tigers, elephants, and other small game. They will afterwards proceed to Bombay, through the Red Sea and Suez canal to Cairo, up the Nile to the first cataract, then return to Port Said, thence to Joppa and the Holy Land, thence to Constantinople, Athens, and through Italy, France, Germany, Russia, Norway, Sweden and Great Britain, after which they will sail for home. They expect to be absent about fifteen months.

---

DR. B. L. HOVEY, of Rochester, Surgeon of the Buffalo, Rochester & Pittsburgh R. R., was elected Fourth Vice-President of the National Association of Railway Surgeons at the annual meeting of that organization, held at Chicago June 28, 1888.

DR. A. G. GUMAER, of this city, is a member of the association.

---

DR. CAROLINE S. RODGERS, of Rochester, has been appointed examining physician to the department for women of the State Industrial School, the first appointment by the State of a woman in the capacity of a physician

